# Goal change and goal achievement for emerging adults across the pilot FAMS-T1D intervention for type 1 diabetes

**DOI:** 10.3389/fcdhc.2024.1297422

**Published:** 2024-04-15

**Authors:** Cynthia A. Berg, Jessica H. Mansfield, Silas B. Boggess, Julia V. Martin, Benjamin Creer, Torri K. Peck, Deborah J. Wiebe, Jonathan E. Butner, Lindsay S. Mayberry

**Affiliations:** ^1^Department of Psychology, University of Utah, Salt Lake City, UT, United States; ^2^Psychological Sciences, University of California, Merced, CA, United States; ^3^Department of Medicine, Vanderbilt University Medical Center, Nashville, TN, United States

**Keywords:** type 1 diabetes, emerging adulthood, goals, intervention, social support, self regulation

## Abstract

**Objective:**

Interventions for emerging adults (EAs) with type 1 diabetes (T1D) focus on goal setting, but little is known about how goal achievement relates to intervention outcomes. We examined how goals change, how goal achievement relates to diabetes outcomes, and identified barriers and facilitators to goal achievement.

**Method:**

EAs with T1D (N=29, *M* age=21.6 years, 57% female) were coached monthly to set a behavioral goal across a 3-month feasibility trial. Coaching notes were qualitatively coded regarding type, complexity, and changes in goals. Goal achievement was measured via daily responses to texts. HbA1c, self-efficacy, diabetes distress, and self-care were assessed pre- and post-intervention.

**Results:**

EAs frequently set food goals (79%) in combination with other goals. EAs overwhelmingly changed their goals (90%), with most increasing goal complexity. Goal achievement was high (79% of days) and not affected by goal change or goal complexity. Goal achievement was associated with increases in self-efficacy and self-care across time. Qualitative themes revealed that aspects of self-regulation and social-regulation were important for goal achievement.

**Conclusion:**

Meeting daily diabetes goals may enhance self-efficacy and self-care for diabetes.

**Practice Implications:**

Assisting EAs to reduce self-regulation challenges and enhance social support for goals may lead to better diabetes outcomes.

## 1 Introduction

Type 1 diabetes (T1D) affects 1.9 million Americans (1) who coordinate food intake and exercise with monitoring blood glucose (BG) and taking insulin. Emerging adulthood is a high-risk time for managing diabetes (2), as emerging adults (EAs) experience high diabetes distress (3) and HbA1c (glycosolated hemoglobin, representing sugar in the blood) (4), with 17% meeting treatment recommendations for HbA1c less than or equal to 7.0% (5).

Emerging adulthood is challenging for diabetes as it involves changes in the social context when self-regulation is not fully developed (6). Successful T1D management involves self-regulation, including setting and achieving goals (7). Self-regulation failures (e.g., forgetting to check BG) create challenges for self-care (8, 9). In the social context, parental involvement declines across emerging adulthood, with such involvement beneficial for those with lower self-regulation (10). Although romantic partners and friends become potential sources of support, they are not uniformly beneficial (11), as EAs may not know how to utilize them for support and they may be unaware of how to be supportive (12). Thus, EAs experience challenges in self-regulation (goal planning, regulating cognition and behavior to facilitate goals) and social-regulation (communication to optimize social support) related to diabetes self-management.

Interventions to assist EAs focus on setting goals (13, 14), one component of self-regulation. For instance, REAL (14) includes a module on goals together with skill building surrounding living with diabetes. Such goal-setting interventions are common in diabetes management (15). We modified a successful intervention developed for adults with type 2 diabetes (16) for EAs with T1D. FAMS (Family/friend Activation to Motivate Self-care)-T1D is an mHealth intervention that helps individuals set diabetes-specific goals and build skills to manage social relationships. Text messages to EAs with T1D facilitate goal monitoring and to a support person (SP; when invited) optimize their support. In the feasibility study with EAs, FAMS-T1D was acceptable, feasible, and pre-post improvements were found in self-efficacy, self-management, and diabetes distress (Mayberry et al., 2024).

Although goal setting and weekly goal management is often central to diabetes interventions (15) and education (17), little is known about the content and complexity of goals and whether changes in goals are linked to goal achievement or intervention outcomes (18). This gap is because the specific goals that individuals set and changes in those goals together with goal achievement are rarely charted across interventions. Observational work has showed that goal planning is associated with better self-care and BG (19). As T1D self-management requires the coordination of multiple behaviors, interventions involving goal setting could engage individuals across time to set diabetes goals that are increasingly complex such that goals regarding BG monitoring (BGM) may be combined with food intake and insulin adjustment. Research on pursuing multiple goals simultaneously has been growing in the goal literature (20, 21), but has not been applied to the challenges of diabetes management.

In the present paper, we conducted an exploratory mixed-methods analysis from a 3- month intervention. Through analyzing coaching notes, we examined how aspects of goals (goal content, change, and complexity) were associated with daily goal achievement and changes in self-efficacy, diabetes distress, self-care and HbA1c across the intervention. We did not have *a priori* hypotheses regarding whether EAs would be able to maintain goal achievement when they changed their goals. The broader goal literature indicates that more complex or difficult goals that are within one’s reach may lead to enhanced goal performance (21). Changes in goals could involve a natural progression of diabetes management to integrate increasing complexity. We predicted that goal achievement would relate to pre-post differences in diabetes outcomes. In addition, we coded mentions of facilitators and challenges in self- and social-regulation to understand how they were involved in goal attainment.

## 2 Materials and methods

### 2.1 Study setting and design

**Figure 1:**
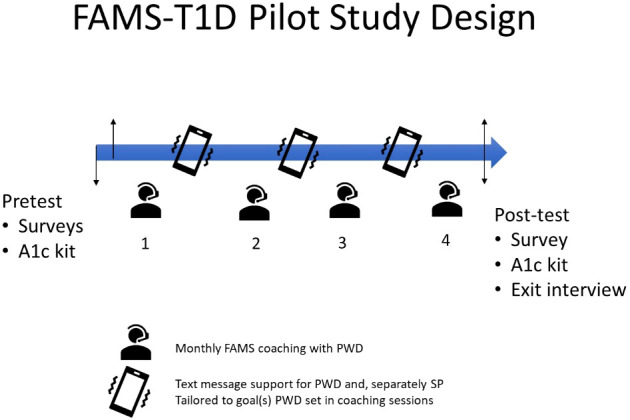
Design of the FAMS-T1D Pilot Study.

This is a secondary, mixed-methods analysis from a pre-post pilot three-month intervention study of EAs with T1D (*N*=29). The intervention included three coaching sessions where goals were set (a fourth session assessed qualitative feedback regarding the intervention), three months of daily text message support, plus completion of pre-post measures (see **Figure 1**).

The FAMS-T1D intervention was adapted from FAMS (16) and involved three components: 1) monthly coaching to set SMART (specific, measurable, actionable, realistic, and time-bound) diabetes-management goals and develop skills to facilitate family/friend involvement for goals, 2) one-way and interactive text messages tailored to the goal, and 3) an option to invite a friend/family member as a SP, who received text messages to increase their support for the person with diabetes.

Coaching sessions occurred by phone with a coach with a Master’s degree in Social Work. In sessions one through three, the EA set a behavioral goal and engaged in coach-selected exercises to build social-regulation skills. Session one included goal setting and the role of others in diabetes self-management. EAs were guided to set personalized goals for managing food (e.g., eat three meals per day for 4 days) or BGM (e.g., test BG before leaving work 4 days a week). Each subsequent session involved discussion of goal progress and an opportunity to adjust or change the goal, followed by coach-directed skill building to enhance social regulation. Each session ended with an agreement to engage a specific person in plans to meet the goal using skills learned in coaching.

The same coach completed all sessions and used the standardized FAMS coaching casenote, entered into REDCap, to capture data on the coaching session (usually immediately, always within 24 hours). In addition to structured items (e.g., goal type) the coach wrote a detailed summary of each session including how the goal was set, what made it easier or more difficult to achieve the goal, and experiences of the EA with friend and family involvement. EAs received text messages (3-4 per week) tailored to their preferred windows of time and type of goal (i.e., food or BGM) and received an interactive message asking if they met their goal each day. Tailoring of both one-way and interactive messages was updated to reflect changes made to goals during each subsequent coaching session.

### 2.2 Participants

Eligible individuals were 18-24 years of age, diagnosed with T1D, taking insulin for at least one year, had a mobile phone, were comfortable sending texts, and could speak and read in English. SPs (if invited) were eligible if they were 18 years of age or older. We excluded participants with limitations that would preclude participation including an intellectual disability, blindness or auditory limitations, or severe mental illness.

We recruited individuals with diabetes from a diabetes clinic in the southwest U.S. Electronic medical record data were used to identify eligible individuals seen in the clinic in the prior six months. An opt-in/opt-out letter describing the study was sent to potential participants. The person with diabetes was contacted via phone or text message to explain the study to those who did not opt-out to confirm eligibility.

### 2.3 Measures

#### 2.3.1 Quantitative

*Goal achievement* was measured by a daily text that EAs received around their bedtime asking “Did you meet your SMART goal today, Monday 6/15? Please reply Yes or No.*”* Goal achievement was the number of responses that a person answered yes divided by the total number of responses that an individual provided. Thus, if an individual did not respond to a text, this was taken into account with the calculation of goal achievement. Any response triggered encouraging automated feedback.

Diabetes outcomes were measured prior to and at the conclusion of the intervention. *Diabetes self-efficacy* was assessed with the 10-item Self-Efficacy for Diabetes Management Scale (22), with EAs rating their confidence in completing diabetes tasks (e.g., “How sure are you that you can manage your diabetes even when you feel overwhelmed”) on a 1= “not sure at all” to 10= “completely sure” scale. We added four items to capture transition issues common among EAs (e.g., “make your doctor’s appointments”). Items were averaged with higher scores indicating more self-efficacy (α=0.80, good). *Diabetes self-care* was assessed with the Self-Care Inventory Revised (23), a 13-item measure assessing how often respondents perform T1D behaviors (e.g., checking blood glucose) on a 1= “never do it” to 5= “always do this as recommended without fail” scale. Items were averaged with higher scores indicating better self-care (α=0.75, acceptable). *Diabetes distress* was assessed with the Problem Areas In Diabetes (PAID) scale (24), a 20-item measure evaluating dimensions of distress on a 0= “not a problem” to 4= “serious problem” scale. Items were summed and transformed into a score ranging from 0-100; higher scores indicated more diabetes distress (α=0.92, excellent). *Hemoglobin A1c* was assessed via mail-in A1c kits completed by EAs and analyzed by CoreMedica Laboratories (Lee’s Summit, MO). The technology used in the kits has been validated against venipuncture (r=.987) (25).

Goal coding. Goal coding data came from the coach’s casenotes of each session. Goals were coded by three of the authors into one of seven categories: Food, BGM, food+BGM, food+insulin, food+exercise, BGM+insulin, and food+BGM+insulin. One coder was used as the criterion coder (CAB), with coders showing excellent agreement with the criterion coder (coder 1 Kappa=1, coder 2 Kappa=.98). Disagreements were discussed and resolved as a group. Complex goals involved more than one goal type (e.g., BGM+insulin) as opposed to only one type (e.g., BGM alone). Coders assessed change in goals across monthly coaching sessions with three codes: no change (same goal across sessions), goal adjustment (goal type remained the same but was adjusted, e.g., goal changed from eating breakfast three days per week to eating breakfast five days per week), and goal change (goal type changed, e.g., from a food goal to a BGM goal). Good agreement was achieved with the criterion coder (Kappa=.70). Disagreements were discussed and resolved by consensus.

#### 2.3.2 Qualitative

From the coaching casenotes, self-regulation and social-regulation challenges and facilitators were coded (see analysis section for greater details).

### 2.4 Data analysis

#### 2.4.1 Data analysis: quantitative and mixed-methods

Descriptives of goal types, goal complexity, and goal change were calculated. Next, links between goal achievement and goal type, complexity, and goal change were analyzed via multi-level modelling in IBM SPSS Mixed (version 27, 2020) (26) to take into account the nested nature of sessions within individuals. Goal session was controlled in all analyses (i.e., session 1, 2, or 3). For analyses involving goal type, we created indicator variables for each type – for instance, a food goal was denoted as 1 when a food goal was set either alone or in combination with another goal. Separate analyses were conducted for food and BGM goals. The indicator variables (e.g., no food goal/food goal) were included as Level 1 within-person predictors. We did not center within-person at Level 1 as the variable is dichotomous and the restricted range of possibilities does not allow for a clear separation of within- and between-person effects (27), thus between-person effects are not interpreted. The mean frequency of goal type across the three time points (1–3) was grand mean centered and included as a Level 2 between-person predictor to account for individual differences. The Level 1 effect reflects the within-person effect, holding the between-person effect constant. Similar analyses were conducted for goal complexity (0=single goal, 1= multiple goal). Next, we performed analyses linking goal change with the subsequent month’s goal achievement (across sessions 1 and 2, then 2 and 3 yielding two time points, note goals were not set in session 4). Next, links between goal achievement across the three months and post-test self-efficacy, self-care, diabetes distress, and HbA1c were examined via multiple regressions, holding constant pre-intervention values such that coefficients can be interpreted as change in the outcome of interest.

#### 2.4.2 Data analysis: qualitative

Coaching notes were analyzed thematically, to identify emerging themes with respect to *self- and social-regulation challenges and facilitators*. First, the first six authors familiarized themselves with the data. Next, the coding team generated codes together, added themes as needed, refined the themes, and named the themes across multiple coding sessions. The coding team then jointly coded the interviews using the generated codebook and discussed until a consensus was reached if different interpretations occurred. Codes were entered into the transcripts via NVivo version 1.3.

## 3 Results

### 3.1 Descriptives and sample characteristics

**Table 1** includes sample descriptives for individuals in the study. The majority of individuals with diabetes invited a SP to participate (70%, n=21), with all but one participating (20/21).

**Table 1:** Sample Characteristics.

Variable	Means (SD)	Ranges
Age	21.54 (1.36)	19.23-24.00
Female	57%	
Education Status		
High school graduate	16.70%	
Some college	50%	
Associate’s degree	16.70%	
Bachelor’s degree	16.70%	
Non-Hispanic White	3.00%	
Duration of diabetes (years)	9.83 (5.49)	1.00-18
HbA1c at baseline	8.21 (1.57)	5.50-12.70
Pump (yes)	73.30%	
CGM	76.70%	

Data are means unless otherwise noted as percentages.

### 3.2 Content of goals and goal change

Food goals were most frequent across all sessions with BGM goals next most frequent (see **Table 2**). Goals involving food (46.7%) or BGM alone (26.7%) comprised the majority of goals in session 1, but complex goals comprised the majority of goals in sessions 2 and 3. Individuals overwhelmingly made changes to their goals between sessions 1 and 2 and sessions 2 and 3, with changes in goal type being most frequent (see **Table 2**).

**Table 2:** Frequencies of Goal Types and Goal Changes Across Sessions.

Goal Type	Session 1	Session 2	Session 3
Food	46.70%	26.70%	34.50%
BGM	26.70%	13.30%	10.30%
Food+BGM	3.30%	13.80%	3.40%
Food+insulin	20%	13.80%	27.60%
Food+exercise	3.30%	24.10%	6.90%
BGM+insulin	0.00%	3.40%	10.30%
Food+BGM+insulin	0.00%	3.40%	6.90%
Goal Changes from prior session			
No Change		6.90%	23.30%
Goal Adjustment		31.00%	20.00%
Goal Type Change		62.10%	53.30%

### 3.3 Goal achievement and links to goal type, complexity, and change

Individuals reported that they achieved their goal the majority of the time (mean= 79%, SD=15% of the days that they responded to daily text messages), with a range from 43% to 100%. Response rate to the question of meeting their goals was high (mean=85%). No within-person effect of goal type (food or BGM) occurred, indicating that goal type did not relate to goal achievement (see **Table 3**). The variance component on the intercept indicated that there was variability across participants in goal achievement. No effect of goal session was found on goal achievement, indicating that goal achievement did not change across the intervention period.

**Table 3:** Multi-level Analysis of Goal Features (type, complexity, change) on Goal Achievement.

	Food Goal	BGM Goal	Complex Goal	Goal Change
	*b (SE)*	*b (SE)*	*b (SE)*	*b (SE)*
Intercept	0.835 (.052)**	.768 (.035)**	.812 (.035)**	.852 (.104)**
Session	.005 (.018)	.004 (.018)	0.016	-.008 (.043)
Within-person	-.067 (.054)	.050 (.049)	-.082 (.041)	-.036 (.042)
Between-person	.287 (.102)**	-.165 (.092)	.103 (.089)	.020 (.08)
Variance Intercept	0.012**	.015**	.017**	0.014

** p < .01.

A similar analysis was conducted to ascertain whether goal complexity (i.e., a goal involving two or more goal types) was associated with goal achievement. No significant associations were found between goal complexity and goal achievement (see **Table 3**).

To assess whether goal change was associated with subsequent goal achievement, a multi-level analysis examined whether goal change across sessions 1 and 2, then 2 and 3 were associated with subsequent goal achievement. No significant effects were found indicating that goal change was not associated with subsequent goal achievement (see **Table 3**).

### 3.4 Goal achievement and pre-post changes in outcomes

Multiple regressions linking goal achievement and change in diabetes outcomes revealed that greater goal achievement across the intervention reported via the texts was associated with change toward higher self-efficacy and self-care. No effects were found for diabetes distress or HbA1c (see **Table 4**). Independent t-tests examining whether outcomes differed by whether a SP participated revealed no significant differences (*p >.7)*. Analyses were also conducted to address whether for those on a CGM, goal achievement was associated with better blood glucose post-intervention controlling for baseline. Fifteen individuals had CGM data available on 70% of the days in a two-week period at baseline and in the last two weeks of the intervention. No significant effect of goal achievement was found on average blood glucose or time-in-range (p >.5).

**Table 4:** Multiple Regressions Predicting Time 2 Outcomes (controlling for Time 1 measures).

	Self-efficacy	Self-care	HbA1c	Diabetes Distress
	*b (SE)*	*b (SE)*	*b (SE)*	*b (SE)*
Intercept	0.745 (1.164)	0.725 (.563)	0.139 (1.228)	-13.08 (12.23)
Pre-intervention	.641**(.146)	.565** (.132)	.896** (.00)	.586** (.136)
Goal Achievement	2.904 (1.346)*	1.180* (.516)	0.552 (1.161)	21.217 (14.671)

*p <.05; ** p <.01.

### 3.5 Self- and social-regulation facilitators and challenges to goal achievement

#### 3.5.1 Self-regulation

Six themes emerged regarding self-regulation, with four oriented toward things that facilitated or accompanied goal achievement (planning ahead to meet the goal, habit, goal was achieved, feeling proud) and two related to barriers (changes to a prior routine, challenges in cognitive regulation) Consistent with the goal of coaching, one theme involved *planning ahead to meet the goal*. Casenotes indicated that one EA mentioned he “found setting an alarm in the morning really helped him plan out a time in the day to meet the goal.” Another mentioned “it was easier on days that she prepared for the next day to meet her goal.” EAs mentioned that the coaching made it so that their *goal was achieved*: participant “met goal easily last month and even expanded the goal to include every time he was at work.” For some, the goal became a *habit* “having a snack when exercising has now become a habit and she has started bringing a snack with her everywhere she goes” and “noted last month’s goal became easier as the month went on, which participant attributed to the action of bolusing becoming more of a habit.” Individuals mentioned that goal achievement made them *feel proud*: “she feels as though she has proved to herself that she can make changes in her health, something she previously thought impossible.”

Two themes emerged regarding challenges in self-regulation that made goal achievement difficult. *Changes to a prior routine* made goal achievement difficult such as travel, working more, having a busier schedule than normal, or sickness: participant “mentioned she and her husband traveled to see family last month, so it made meeting her goal difficult because she wasn’t preparing dinner and didn’t always know when they would be eating.” EAs mentioned a wide range of *challenges in cognitive regulation* including feeling unmotivated to deal with their diabetes. A participant “also rated her confidence low because she ‘doesn’t care’, stating she has never been able to find anything that would motivate her.” Casenotes mentioned forgetting to engage in goal pursuit, e.g., “goal was difficult last month because he often forgot about it.”

#### 3.5.2 Social-regulation

Six themes involved social regulation with two related to facilitators (receiving support from their selected SP, expand the support beyond what they solicited from the SP) and four to challenges (unhelpful behavior, others uninvolved, saw self as independent, social constraints). Consistent with the goals of the intervention, individuals reported *receiving support from their selected SP*. For example, a participant “noted her husband trusts her to take care of her BG and to ask for help and stated this dynamic has been really freeing and helped change her relationship with diabetes.” A participant “also noticed that her mother cooked at home more and would calculate the exact carbs in a serving of the meal. Participant found this extremely helpful and was impressed with her mother’s support.” In addition, EAs described that as they were trying out their strategies they began to *expand the support beyond what they solicited from the SP* with some being surprised by their level of support. For example, “Participant said her manager [at work] was really understanding and made patient feel welcome when she explained having T1D to him.”

Four challenges emerged with respect to social barriers in achieving goals. Individuals experienced *unhelpful behavior* from others, including obstructive behavior, individuals making unkind comments, or being overinvolved or involved in a controlling manner. One participant mentioned multiple examples when her father showed that he does not understand T1D (e.g., asking if she needs insulin when she was low or “made her feel embarrassed for having T1D saying it was gross that she took insulin at the table while out to eat.”) Some individuals mentioned that others were *uninvolved*, despite the EA asking them to become involved. One EA “told her parents about her goal but was disappointed when they didn’t check in about it when she visited them.” Sometimes, this uninvolvement may have arisen as the EA mentioned that they saw themselves as *independent* and not in need of assistance. When asked about involving others in the goal, one EA “mentioned that it was nice to have support, but it didn’t always feel relevant since he spends so much time alone and cares for his health on his own.” EAs mentioned that *social constraints* made diabetes more challenging such as feeling uncomfortable talking to their boss: participant “stated that he needed to have a talk with his boss but clearly felt awkward committing to it.”

## 4 Discussion and conclusion

### 4.1 Nature of goals and goal achievement

The content of T1D goals involved a mixture of behaviors that were complex and changing across the intervention. Initially goals largely dealt with food management, but over time more frequently combined food with insulin administration and exercise and BGM. The prominence of food goals was surprising as prior work (19) indicated that goals dealing with BG testing and keeping BG in control were most frequent. The salience of food goals is important clinically, given the frequent emphasis on BGM in T1D. Consistent with the combination of behaviors required for self-management of T1D (e.g., checking blood glucose and managing food and exercise), goals were often complex and increasingly so across the intervention with goals most commonly changing.

EAs were able to change their goals toward more complex goals without a negative impact on goal achievement. Goal achievement as assessed by daily self-report was high across the intervention. Qualitative coding of post-intervention interviews regarding the intervention revealed that synergy between the support provided by the coach for goals together with text messages may have contributed to high goal achievement (Mayberry et al., 2024). The lack of relationship between goal change or goal complexity and goal achievement suggests that EAs can alter their goals to increasingly take on the complexity of diabetes management with the assistance of a coach and text support.

Goal achievement was associated with increases in self-efficacy and self-care across the intervention. These links between daily goal achievement and intervention outcomes point to the value of examining goal achievement in interventions focused on goal setting (13) Qualitative analyses indicated that many young adults mentioned feeling proud to meet health goals. Goal achievement was not associated with increases in diabetes distress across the intervention, suggesting that EAs can set increasingly complex goals without increasing distress. The lack of association between goal achievement and diabetes distress may mean that the effort that comes with achieving one’s goal may raise awareness of the challenges of diabetes management for some. With more time, however, as the behavior becomes established as a habit, we would expect distress to decline. The lack of association between goal achievement and HbA1c may indicate that a longer intervention is needed to show effects on HbA1c, which represents blood glucose across a three- to four-month period.

### 4.2 Facilitators and challenges to goal achievement

Qualitative analyses of the coaching notes revealed facilitators and challenges to goal achievement that involved self-regulation and social-regulation. Goal planning to prepare in advance how to optimize goal achievement is an important facilitator. A prominent barrier to goal achievement was the uncertain schedules that often characterize EAs. Travel, changes in schedules, unusual busy schedules at work, or breaks from school were challenges to sticking with their goal. Coaching to anticipate these changes and plan around them will be important to incorporate into clinical practice (17) and future interventions. These changes to routine may be especially problematic for EAs given they commonly mentioned challenges in self-regulation (e.g., remembering to test) that may be indicative of broader self-regulation failures experienced by EAs (7, 9).

The qualitative analyses revealed the value of having a SP enrolled in the study. The SP was viewed as beneficial and the EA built on this experience by expanding support seeking to others in their network. The value of such support was often surprising to EAs. Type 1 diabetes is most frequently viewed by adults as their own illness (28) and they may not want to burden others. The FAMS-T1D intervention provided strategies for the person with diabetes as well as their SP regarding how to engage in supportive and less unhelpful ways.

The results should be interpreted in the context of limitations. A primary limitation is that we relied on coding of coaching notes, rather than transcripts of coaching sessions. Additional research verifying the coaching notes with what participants say in their own words is needed. The pilot nature of this intervention translated into a small sample size, which limited our ability to find significance in some of our statistical tests. We had only three coaching sessions; more fine-grained analyses examining goal achievement shortly after goal change might better reveal the time course of goal change and achievement. A fully powered randomized clinical trial is underway to address these limitations.

### 4.3 Practice implications

The results have implications for clinical practice. Integrating tracking of goal progress into clinical practice (e.g., through diabetes educator sessions or EHR) could assist EAs to maintain goal pursuit for diabetes management. Helping EAs anticipate times of uncertainty (busy times at work, vacations) characteristic of emerging adulthood may be crucial to maintaining goal pursuit. Also, encouraging EAs to use their support network may facilitate goal achievement.

## Data availability statement

The raw data supporting the conclusions of this article will be made available by the authors, without undue reservation.

## Ethics statement

The studies involving humans were approved by University of Utah Institutional Review Board. The studies were conducted in accordance with the local legislation and institutional requirements. The participants provided their written informed consent to participate in this study.

## Author contributions

CB: Conceptualization, Data curation, Formal Analysis, Funding acquisition, Methodology, Project administration, Resources, Supervision, Writing – original draft, Writing – review & editing. JHM: Data curation, Project administration, Writing – review & editing. SB: Data curation, Writing – review & editing. JVM: Data curation, Writing – review & editing. BC: Data curation, Writing – review & editing. TP: Data curation, Writing – review & editing. DW: Conceptualization, Methodology, Project administration, Writing – review & editing. JB: Formal Analysis, Writing – review & editing. LM: Conceptualization, Funding acquisition, Methodology, Project administration, Writing – review & editing.
